# Use of virtual care by infectious disease specialists in Canada: A national survey

**DOI:** 10.1017/ash.2022.246

**Published:** 2022-06-30

**Authors:** Philip W. Lam, Ilan S. Schwartz, Richard J. Medford

**Affiliations:** 1 Division of Infectious Diseases, Sunnybrook Health Sciences Centre, Toronto Ontario, Canada; 2 Department of Medicine, University of Toronto, Toronto, Ontario, Canada; 3 Division of Infectious Diseases, Department of Medicine, Faculty of Medicine and Dentistry, University of Alberta, Edmonton, Alberta, Canada; 4 Division of Infectious Diseases & Geographic Medicine, University of Texas Southwestern Medical Center, Dallas, Texas, United States; 5 Clinical Informatics Center, University of Texas Southwestern Medical Center, Dallas, Texas, United States

## Abstract

**Objective:**

The aim of this study was to characterize the type and extent of virtual care use among infectious disease specialists in Canada, with a focus on the clinical factors that influence the decision to provide virtual versus in-person care.

**Methods:**

Infectious disease physicians practicing in Canada were invited to complete a survey regarding their experiences with virtual care. The survey included 14 vignettes depicting new outpatient and post–hospital-discharge referrals. Participants were asked to select which (if any) virtual care modalities they would feel comfortable using and to specify a reason if they did not feel comfortable providing care virtually. Machine learning and natural language processing techniques were used to identify themes.

**Results:**

In total, 57 infectious disease physicians completed the survey. Respondents reported devoting 36.5% (SD, 18.4%) of their infectious disease practice to outpatient care, with 44.2% (SD, 23.2%) of it being delivered virtually. Respondents were more comfortable providing virtual care to post–hospital-discharge referrals who had been seen by an infectious disease physician compared to new outpatient referrals. When respondents were not comfortable with using any virtual care modality, the following common themes emerged: the need for physical examination, the importance of establishing a therapeutic relationship, the need for additional in-person tests or diagnostics, and patient counselling.

**Conclusion:**

This study provides a glimpse into the current state of virtual care use in Canada and some of the major themes that affect decision making for virtual versus in-person care.

The coronavirus disease 2019 (COVID-19) pandemic has facilitated the rapid uptake of virtual care in primary and subspecialty care. Although the implementation of virtual care has been described in certain infectious disease settings,^1–3^ little is known about the specific clinical factors that influence infectious disease specialists to select a virtual care modality over an in-person assessment. Understanding the case types that are most amenable to virtual care can inform ambulatory care practice as well as other models where virtual care has been implemented, including inpatient consultation services and antimicrobial stewardship programs.

In this study, we characterized the type and extent of virtual care use among infectious disease specialists in Canada, with a focus on the specific clinical factors that influence the decision to provide virtual versus in-person care.

## Methods

We conducted an open survey of infectious disease physicians practicing in Canada. The survey consisted of 23 nonrandomized questions equally distributed over 5 pages. Questions were multiple choice, Likert scale, and open-ended questions (Supplementary Material).

The first section of the survey collected information pertaining to demographics, practice setting, and past and current use of 3 different virtual care modalities in the outpatient setting: telephone, video conferencing platform, or physician-to-physician electronic consultation. We chose to study virtual care use in the outpatient setting because it has been understudied and poses additional challenges compared to inpatient virtual consultations, for which physical examination findings and investigations are generally more accessible.

The second section of the survey consisted of 14 adult case vignettes. Among them, 7 vignettes depicted outpatient referrals for new consultations of patients not previously seen by an infectious disease physician, and 7 vignettes depicted follow-up of patients discharged from hospital 2 weeks prior who had been seen by an infectious disease physician during hospitalization. The topics within the vignettes were chosen based on an informal survey of 15 infectious disease physicians regarding the most common syndromes encountered in outpatient practice. For each vignette, participants were asked to select which (if any) of the 3 virtual care modalities (ie, telephone, video conferencing platform, or physician-to-physician electronic consultation) they would feel comfortable using to provide care. Although physician-to-physician electronic consultations are technically devoid of patient interaction, we opted to include this option in the vignette because it represents a realistic option of care that may be used in some regions of Canada where direct access to an infectious disease specialist is difficult. Participants were allowed to choose >1 virtual care modality. If the participant indicated that they did not feel comfortable providing virtual care, they were asked to provide a reason. For all vignettes, participants were asked to assume that there were no patient, technologic or remuneration barriers to providing virtual care.

The survey content was developed by the study authors (P.W.L., I.S.S., and R.J.M.). Additional feedback and usability testing were carried out by 3 infectious disease physicians external to the study team. No remuneration was provided for survey participation.

### Data sources and extraction

An e-mail invitation to participate in the survey was sent by the Association of Medical Microbiologists and Infectious Diseases (AMMI) Canada to infectious disease physician members on November 24, 2021. At the time of the survey, 443 infectious disease physicians were members of AMMI. A reminder e-mail was sent on December 8, 2021. An additional email invitation was sent to the program or division director of each infectious diseases academic center across the country inviting further participation. The survey closed on January 15, 2022. The survey was administered using the Qualtrics XM online survey platform. Respondents were only allowed to answer the survey once. Unique survey responses were tracked by internet protocol address and by an internet browser cookie.

### Statistical and thematic analysis

Only completed surveys were included in the analysis. We used the geolocation associated with each Canadian internet protocol address as a surrogate of practice location. For each question, the frequency and proportion of respondent answers were calculated and tabulated. To identify themes within the free-text responses of the survey-related questions, we employed multiple, unsupervised, machine learning and natural language processing techniques to identify clusters and potential topics (topic modeling). Using the gensim library in Python version 3.9.2 software, we applied an algorithm called Latent Dirichlet Allocation (LDA) to group representative words within the free-text responses of the vignette sections (ie, respondents were asked to specify why they were not comfortable using virtual care) and general comment section into word clusters. We subsequently analyzed all word clusters to identify the content of each topic. We trained the LDA model to identify an optimal topical coherence score that evaluated the maximum semantic correlation among high-scoring, frequent words within topics. We ultimately chose a total of 6 topics for section 1, 3 topics for section 2, and 3 topics for the general comment section based on ideal coherence scores. An individual without insight into the study design or topic modeling analysis labeled the topics using the top 20 most frequently used words, which were ranked by weight.

The study received approval from the institutional ethics review board at Sunnybrook Health Sciences Centre.

## Results

In total, 57 infectious disease physicians completed the survey. Among them, 25 respondents (43.9%) were aged 30–40 years; 18 respondents (31.2%) were female; and 42 respondents (73.7%) practiced in an academic setting. Respondents were distributed across the country as follows: 6 (10.5%) from Pacific Region (British Columbia), 20 (35.0%) from Prairie Provinces (Alberta, Saskatchewan, Manitoba), 28 (49.1%) from Central Region (Ontario, Quebec), and 2 (3.5%) from Atlantic Provinces (New Brunswick, Nova Scotia, Prince Edward Island, Newfoundland, and Labrador).

### Virtual care utilization

On average, respondents reported devoting 36.5% (SD, 18.4%) of their infectious diseases practice to outpatient care. Before the COVID-19 pandemic, 7.9% (SD 9.5%) of respondents’ outpatient practice was delivered virtually. At the time of the survey (November 2021), 44.2% (SD, 23.2%) of respondents’ outpatient practice was virtual, with an anticipated decrease to 32.3% (SD, 18.5%) after the pandemic. Also, 50 respondents (87.7%) had not received formal training in providing virtual care. Of the virtual care modalities assessed (ie, telephone, video-conferencing platform, physician-to-physician e-consultation), 47 respondents (82.5%) reported using telephone communication most or all of the time. A large proportion of respondents never used a video-conferencing platform or physician-to-physician e-consultation (25 of 57, 43.9% and 31 of 57, 54.4% respectively).

### Case vignettes

**Fig. 1.**
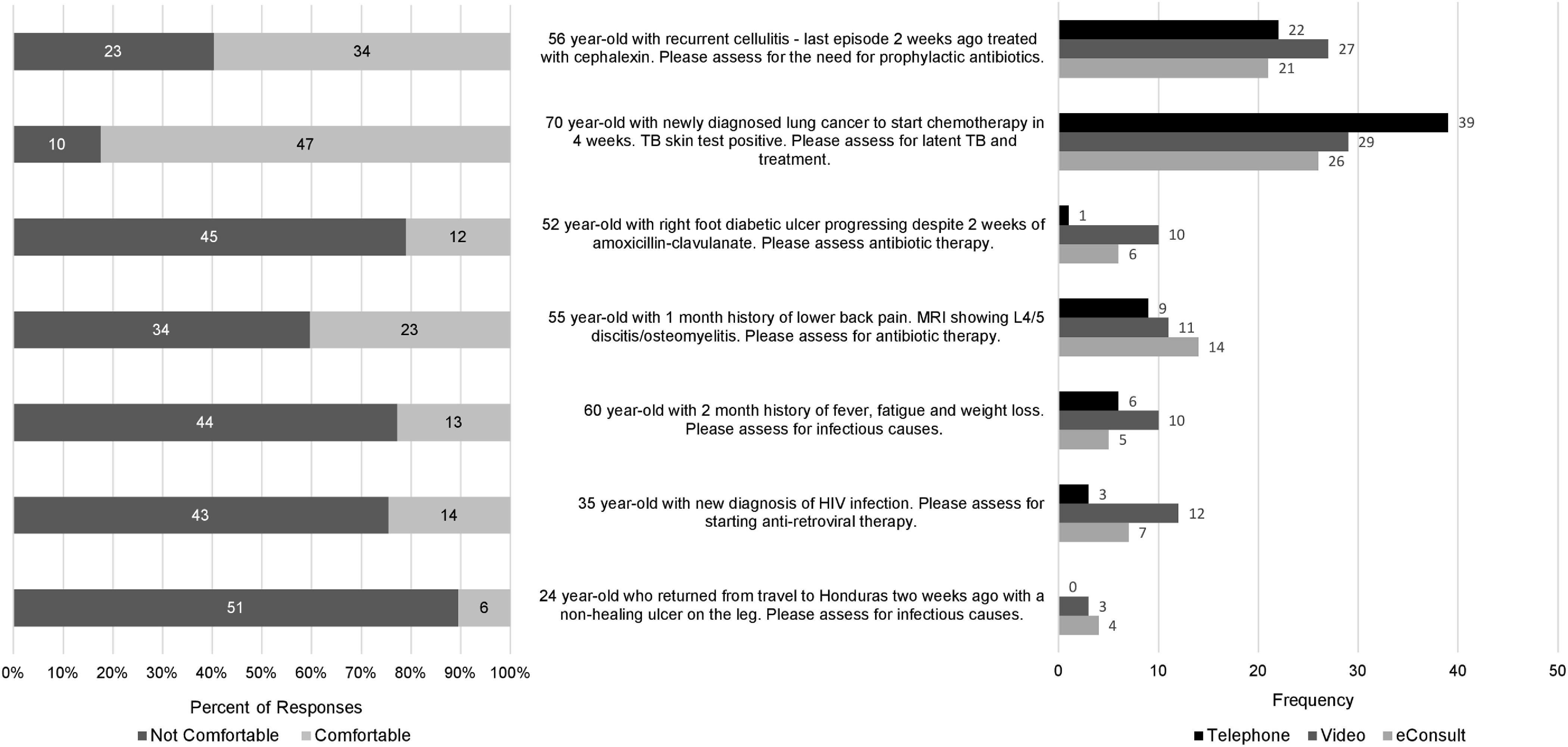
Respondent comfort level and preferred virtual care modality in vignettes representing new outpatient referrals.

**Fig. 2.**
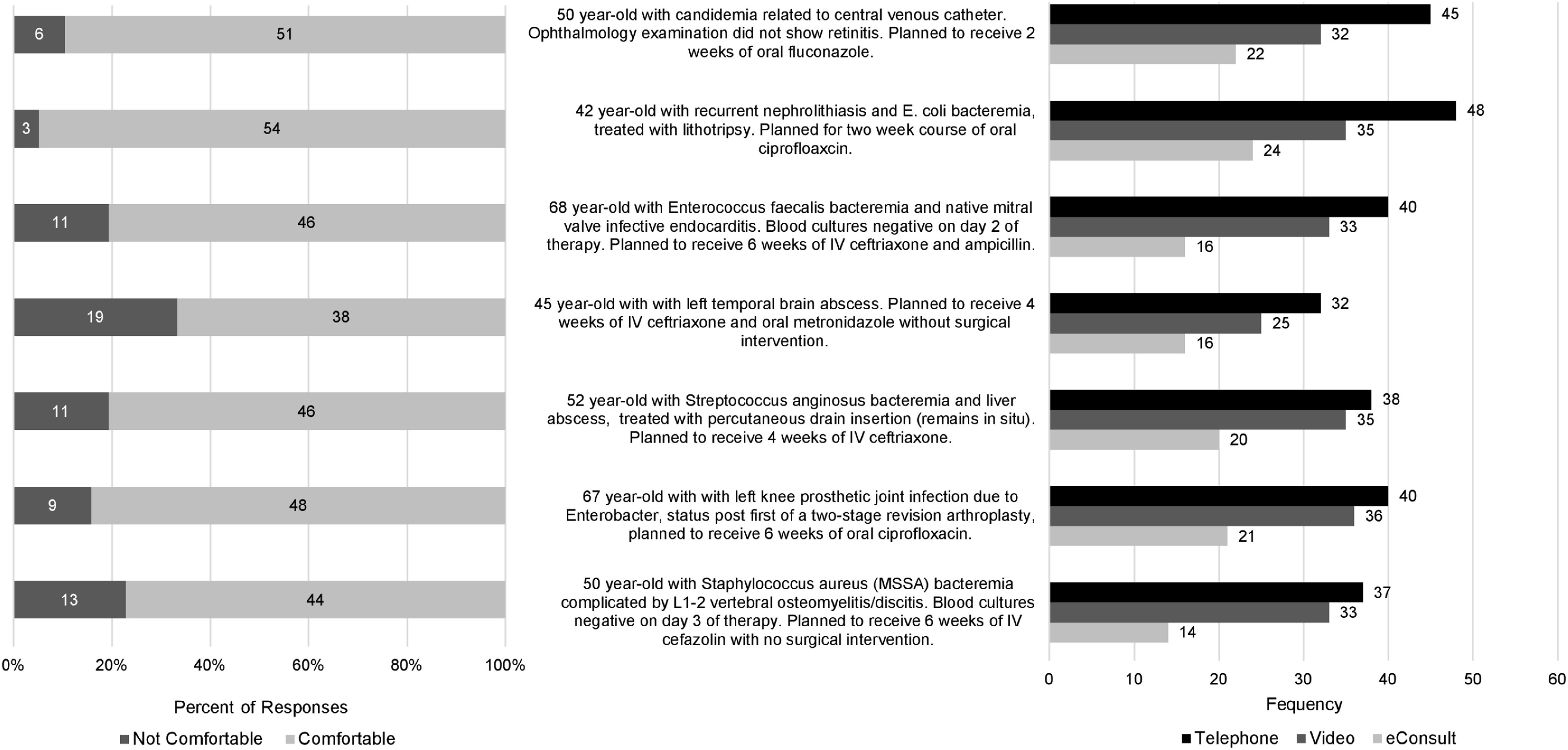
Respondent comfort level and preferred virtual care modality in vignettes depicting follow-up referrals after hospital discharge.

Participant responses to the vignettes are outlined in Figures 1 and 2. In general, respondents were more comfortable providing virtual care to post–hospital-discharge patients who had been seen by an infectious disease physician compared to new referrals. Use of a video conferencing platform was generally preferred over telephone in the vignettes representing new patient referrals, whereas telephone was preferred for vignettes depicting post–hospital-discharge patient referrals. Of the 7 vignettes depicting new patient referrals (Fig. 1), respondents were most comfortable providing virtual care in a referral for latent tuberculosis management and least comfortable in a referral for a nonhealing ulcer in a patient returning from travel. Of the 7 vignettes depicting post–hospital-discharge patient referrals (Fig. 2), respondents were most comfortable providing virtual care in a patient with nephrolithiasis and *E. coli* bacteremia, and least comfortable in a case of a brain abscess that was treated medically.

### Thematic analysis

Thematic analyses were performed on 253, 73 and 42 free-text responses in the new outpatient referral vignettes, post–hospital-discharge vignettes, and general comments sections respectively. The generated clusters of responses and identified themes are outlined in Table 1. More themes were identified for vignettes depicting new patient referrals as opposed to those depicting post–hospital-discharge follow-up. We identified the following common themes from responses to the vignettes (why respondents were not comfortable assessing a patient virtually): the need for physical examination, the importance of establishing a therapeutic relationship, the need for additional in-person tests or diagnostics, and patient counselling. Themes identified from the general comments included the differentiation between new versus follow-up patients, the patient and technological factors that affect virtual care provision, and how the determination of virtual care is often case specific.

**Table 1.** Thematic Analysis of Free Text Responses From Survey Respondents

Cluster	Theme	Frequency, %	Keywords, Ranked by Weight
**Section 1 vignettes: New patient referrals.** *“You indicated that you are not comfortable with any virtual care modality. Please explain why:”*			
1	Thorough physical examination needed	19.6	Need, physical_exam, exam, examination, examine, etc, skin, detailed, biopsy, ulcer, kind_consultation, important_piece, probe, bone, infection, broad, node, spleen, complete, pt
2	Establishing a relationship	19.0	Patient, require_physical_exam, person, need, consult, new, relationship, history, long_term, ensure, physician, well, examine, start, think, physically, follow, meet, evaluate, infectious
3	Additional in-person diagnostics needed	18.3	Need, assessment, person, require, care, thorough, ulcer, comfortable_good_patient, patient, biopsy, look, neurological, assess, physical_exam, perform, physical_examination, infection, therapy, clinical, etc
4	Physical examination exceeds what can be done virtually	17.8	Need, physical_exam, cellulitis, sample, diagnosis, recurrent, important, examine, culture, confirm, collect, heal, stasis, condition, collection, proper, take, id, body, debridement
5	Patient counselling	14	Physical_exam, appropriate, biopsy, ie, require, clinician, counsel, evaluate, culture, need, know_know, certainly_provide, adequately_train_virtual_care, require_sit_home_day, government_half, hypothesis_generation, ass_consultation, incomplete_process, important_prompt, incomplete_advice
6	Value of face-to-face meeting	11.4	Need, person, visit, physical_examination, establish, physical, face, video, patient, initial, non, tb, treatment, assess, active, rule, importance, important, photo, come
**Section 2 vignettes: Post–hospital-discharge follow-up.** *“You indicated that you are not comfortable with any virtual care modality. Please explain why:”*			
1	Need to examine for complications	49.6	Need, exam, examine, patient, physical_examination, assess, infection, look, drain, relapse, neurological, phone, physical_exam, potential_complication_occur_pick, specialist_know, require_job, early_present, reason, well, medicolegal
2	Degree of trust with referring physician	28.8	Patient, assessment, physician, clinical, source, phone, refer, depend, appreciation, variation, w, detect, ensure, happen, E, review, trust, sufficient, okay, probably
3	Patients receiving outpatient parenteral antibiotic therapy require additional support	21.6	Clinic, need, home, lab, leave, patient, med, visit, assess, person, pharmacy, building, day, pick, come, therapy, IV, discharge, time, family
**General comments.** *“Do you have any general comments related to how virtual care is used in your outpatient infectious disease practice?”*			
1	Follow-up visit versus in-person visits	68.1	Patient, virtual_care, person, video, physician, phone, follow, followup, use, work, think, appointment, id, case, consultation, exam, physical, practice, time, care
2	Technologic, patient, and geographic challenges with virtual care	17.5	Difficult, patient, person, set, phone, definitely, use, concern, clinic, need, video, consult, telephone, like, time, community, technical, relate, image, prefer
3	Case dependent	14.4	Virtual_care, care, think, consultation, case, person, patient, comment, plan, management, ID, etc, work, find, doctor, relationship, new, telephone, like, time

## Discussion

In keeping with other medical specialties, use of virtual care within the field of infectious diseases has increased significantly during the pandemic and will likely be a part of outpatient practice after the pandemic. To our knowledge, this is the first study to characterize virtual care use among infectious disease physicians across Canada in the outpatient setting. More importantly, this study is the first to explore the clinical factors that influence the decision to provide virtual versus in-person care. These findings illustrate the perceived limitations of virtual care in the ambulatory care setting and may inform other virtually delivered care models within the field of infectious diseases, such as antimicrobial stewardship prospective audit and feedback, where decisions on antibiotic appropriateness or duration may be dependent on accurate physical examination findings.

Despite respondents delivering a sizable proportion of outpatient practice through a virtual modality, most infectious disease physicians in this survey have not received formal training in providing virtual care. We suspect that this finding reflected the rapid adoption of virtual care as a necessity to maintain continuity of care during the pandemic. As the use of virtual care becomes an accepted modality in healthcare, physicians will need to ensure proficiency in skills unique to virtual care. A framework of telehealth skills has been developed by the Association of American Medical Colleges,^4^ and incorporation of these elements into residency training curricula^5^ will be essential to ensure current and future physicians are competent in providing virtual care upon entering independent practice.

The use of case vignettes in our study provides a glimpse into the major clinical factors that shape a clinician’s decision to provide virtual versus in-person care. The general comfort level in providing virtual versus in-person care varied significantly depending on the content provided in each vignette. We were able to identify common themes using topic modelling. Not surprisingly, the need for physical examination was a commonly cited reason for preferring in-person assessments. Although some aspects of the physical examination can be reproduced virtually,^6^ tactile characteristics (eg, warmth, induration, and fluctuance) may not be easily ascertained virtually. The added value of an in-person consultation was identified by Canterino et al,^7^ who published their experiences of converting an inpatient infectious disease consultation service from in-person to virtual. In their survey of participating infectious disease consultants, 87% felt there were clinical situations where a face-to-face evaluation was necessary, specifically in cases of skin and soft-tissue syndromes, endovascular infection, and unexplained febrile illness.^7^ These findings are congruent with our vignettes. Respondents were most uncomfortable (>75%) providing virtual care to those with skin and soft-tissue syndromes, undifferentiated illnesses, and those who had not responded to empirical antibiotic therapy.

Respondents in this survey indicated that a video platform modality was generally preferred over telephone in vignettes describing new patient referrals. We suspect this preference, in addition to allowing for an additional degree of physical examination, reflects the view that use of video conferencing technology helps to strengthen a therapeutic relationship with the patient compared to a telephone visit. One systematic review of studies examining telephone versus video conferencing modality found that provider outcomes such as diagnostic accuracy and readmission rates improved with video conferencing technology, but patient satisfaction was similar.^8^

This study had several limitations. This survey was conducted in Canada, where the regulation and remuneration for virtual care services are determined provincially and may differ from other countries. With a small sample size, this study was underpowered to assess provincial differences in virtual healthcare use. Secondly, the response rate for this survey was suboptimal and may have been influenced by general fatigue among infectious disease physicians during the pandemic. The number of Canadian infectious disease consultants who provide ambulatory care is unclear, so the response rate could not be calculated. Similarly, the estimates of virtual care use in this survey may be overestimated because of response bias. Thirdly, although the vignettes asked respondents to assume there were not patient-specific, technology-related, or remuneration-related barriers to providing virtual care, we could not definitively control for these factors in their responses. We suspect that respondents’ comfort level in using telemedicine is deeply rooted in prior virtual care experiences within their healthcare setting, and separating these factors is challenging. In addition, the vignettes did not account for pediatric cases of infection, which may introduce additional complexities into the determination of virtual versus in-person care. Finally, this survey has provided a snapshot of outpatient virtual care use in the country, which will likely evolve over time based on other factors, such as the COVID-19 pandemic, and changes to healthcare remuneration for virtual care services.

The field of infectious diseases encompasses a wide range of syndromes and patient populations. Given the high complexity of patients seen by infectious disease physicians,^9^ and strong interplay between infection, the social determinants of health, and disparities in access to virtual care technology,^10–12^ a one-size-fits-all approach to determining virtual care appropriateness is unlikely. Further research is needed to determine the impact of virtual care services on clinical outcomes in the ambulatory setting.^13^ This study provides a glimpse into the current state of virtual care use in Canada and some of the major themes that affect decision making for virtual versus in-person care.

## Supplementary material

For supplementary material accompanying this paper visit https://doi.org/10.1017/ash.2022.246. click here to view supplementary material
