# Supplementary material for: Use of virtual care by infectious disease specialists in Canada: A national survey
Source: Antimicrob Steward Healthc Epidemiol. 2022 Jun 30;2(1):e106. doi: 10.1017/ash.2022.246 (PMC9726522; doi:10.1017/ash.2022.246)
Supplement: Supplementary file 1 [file S2732494X22002467sup001.docx]

ID Virtual Care Survey

Start of Block: Study Information and Consent

**Use of Virtual Care in Infectious Diseases - National Survey**
 
You are invited to participate in a research study being conducted at Sunnybrook Health Sciences Centre. You have been contacted specifically because you have been identified as an Infectious Diseases physician through the Association of Medical Microbiology and Infectious Disease Canada membership list.
 
The purpose of this study is to gain insight into how Infectious Diseases physicians are using virtual care in their outpatient practice across the country. We hope to learn about the various modalities that are being used and how its use varies depending on different clinical scenarios.
 
The study consists of a confidential, online, web-based survey. Survey questions focus on the use of virtual care in your outpatient practice, and a series of clinical vignettes assessing your comfort level in managing various infectious diseases syndromes using virtual care. It will take about 7 minutes to complete. The survey will be open for a four week period and the invitation will be sent to approximately 400 infectious disease physicians across Canada.

You will not be compensated for participating in this study. Your participation in this study will help specialists nationwide gain insight into how virtual care is used for the management of infectious diseases. Once data analysis is complete, we plan to disseminate our findings in the form of a publication. No identifying information will be included in the final publication. Any information that you provide is confidential and any personal information will be de-identified. All data will be summarized, and no individual person will be able to be identified from these summarized results. The data, with no personal identifiers, collected from this study will be maintained on a password-encrypted computer database. Additionally, the data will be electronically archived after completion of the study, maintained for 10 years and then destroyed. This survey uses Qualtrics Core XM, which is a Canadian research software company. All communications to/from the Qualtrics servers are encrypted using transport layer security.


Participating in this study is your choice (voluntary). You have the right to choose not to participate, or to stop participating in this study at any time. You have the right to ask questions and to receive answers throughout this study. If you prefer not to submit your data through Qualtrics, or should you have any questions about the study, please contact the person in charge of this study (Philip Lam - philip.lam@sunnybrook.ca).

If you have questions about your rights as a research participant or any ethical issues related to this study that you wish to discuss with someone not directly involved with the study, you may call the Chair of the Sunnybrook Research Ethics Board at (416) 480-6100 ext. 88144.
 
**Documentation of Informed Consent**
 
By completing this form, I confirm that:

This research study has been fully explained to me and all of my questions answered to my satisfaction

I understand the requirements of participating in this research study

I have been informed of the risks and benefits, if any, of participating in this research study

I have been informed of the rights of research participants

- I agree to participate in this study. (1)
- I do not wish to participate. (2)

Skip To: End of Survey If Use of Virtual Care in Infectious Diseases - National Survey   You are invited to participate in... = I do not wish to participate.

End of Block: Study Information and Consent

Start of Block: Part I: General Information

Q1 What is your age?

- 30-40 (2)
- 40-50 (3)
- 50-60 (4)
- 60-70 (5)
- Over 70 (6)
- Prefer not to say (7)

Q2 How would you describe your gender?

- Male (1)
- Female (2)
- Non-binary / third gender (3)
- Prefer not to say (4)

Q3 How long have you been practising as an Infectious Diseases physician?

- Less than 5 years (1)
- 5 - 10 years (2)
- 10 - 20 years (3)
- Greater than 20 years (4)
- Prefer not to say (5)

Q4 Which of the following best describes your practice setting? (check all that apply)

- Community (1)
- Academic (2)

End of Block: Part I: General Information

Start of Block: Part II: Virtual Care Practices

Q5 What percentage of your Infectious Diseases practice is devoted to outpatient care?

|  | 0 | 10 | 20 | 30 | 40 | 50 | 60 | 70 | 80 | 90 | 100 |
| --- | --- | --- | --- | --- | --- | --- | --- | --- | --- | --- | --- |

| 1 () | 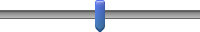 |
| --- | --- |

Q6
In the two following instances, what percentage of your Infectious Diseases outpatient practice has been virtual?

By virtual, we mean: telephone, video-conferencing platform, or physician-to-physician e-consultation.

|  | 0 | 10 | 20 | 30 | 40 | 50 | 60 | 70 | 80 | 90 | 100 |
| --- | --- | --- | --- | --- | --- | --- | --- | --- | --- | --- | --- |

| Practice before COVID-19 pandemic () | 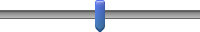 |
| --- | --- |
| Current practice () | 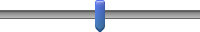 |
| Anticipated practice in future post-pandemic () | 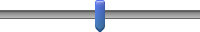 |

Q7 Have you received any formal training in providing virtual care?

- Yes (1)
- No (2)

Q8 Of the patients where virtual care is provided, how frequently do you use each of the following virtual care modalities?

|  | Never (1) | Sometimes (2) | About half the time (3) | Most of the time (4) | Always (5) |
| --- | --- | --- | --- | --- | --- |
| Telephone (1) |  |  |  |  |  |
| Video-conferencing platform (2) |  |  |  |  |  |
| Physician-to-physician e-consultation (3) |  |  |  |  |  |

End of Block: Part II: Virtual Care Practices

Start of Block: Part III: Outpatient Virtual Care Vignettes

Q9 Please consider the following **outpatient referrals for patients who have not been assessed by an Infectious Diseases physician**. For the purposes of the following questions, assume that there are no patient-specific or technology-specific barriers to accessing or administering care virtually. Unless otherwise specified, assume that the referring diagnosis has been confirmed and the accompanying microbiology and investigations are available. Please assume that the remuneration for services provided virtually are identical to those provided in-person.

For each of the following vignettes, please select the modalities which you be comfortable using to assess and manage the patient during the first clinical encounter. (Check all that apply)

|  | Telephone (1) | Video-conferencing platform (2) | Physician-to-physician e-consultation (3) | Not comfortable with any virtual care modality (4) |
| --- | --- | --- | --- | --- |
| Case 1: 56 year-old with recurrent cellulitis – last episode 2 weeks ago treated with cephalexin. Please assess for the need for prophylactic antibiotics. (1) |  |  |  |  |
| Case 2: 70 year-old with newly diagnosed lung cancer to start chemotherapy in 4 weeks. TB skin test positive. Please assess for latent TB and treatment (2) |  |  |  |  |
| Case 3: 52 year-old with right foot diabetic ulcer progressing despite 2 weeks of amoxicillin-clavulanate. Please assess antibiotic therapy. (3) |  |  |  |  |
| Case 4: 55 year-old with 1 month history of lower back pain. MRI showing L4/5 discitis/osteomyelitis – please assess for antibiotic therapy. (4) |  |  |  |  |
| Case 5: 60 year-old with 2 month history of fever, fatigue and weight loss. Please assess for infectious causes. (5) |  |  |  |  |
| Case 6: 35 year-old with new diagnosis of HIV infection. Please assess for starting anti-retroviral therapy. (6) |  |  |  |  |
| Case 7: 24 year-old who returned from travel to Honduras two weeks ago with a non-healing ulcer on the leg. Please assess for infectious causes. (9) |  |  |  |  |

Display This Question:

If For each of the following vignettes, please select the modalities which you be comfortable using... = Case 1: 56 year-old with recurrent cellulitis – last episode 2 weeks ago treated with cephalexin. Please assess for the need for prophylactic antibiotics. [ Not comfortable with any virtual care modality ]

Case 1: *56 year-old with recurrent cellulitis – last episode 2 weeks ago treated with cephalexin. Please assess for the need for prophylactic antibiotics.*


You indicated that you are not comfortable with any virtual care modality. Please explain why:

________________________________________________________________

________________________________________________________________

________________________________________________________________

________________________________________________________________

________________________________________________________________

Display This Question:

If For each of the following vignettes, please select the modalities which you be comfortable using... = Case 2: 70 year-old with newly diagnosed lung cancer to start chemotherapy in 4 weeks. TB skin test positive. Please assess for latent TB and treatment [ Not comfortable with any virtual care modality ]

Case 2: *70 year-old with newly diagnosed lung cancer to start chemotherapy in 4 weeks. TB skin test positive. Please assess for latent TB and treatment.*


You indicated that you are not comfortable with any virtual care modality. Please explain why:

________________________________________________________________

________________________________________________________________

________________________________________________________________

________________________________________________________________

________________________________________________________________

Display This Question:

If For each of the following vignettes, please select the modalities which you be comfortable using... = Case 3: 52 year-old with right foot diabetic ulcer progressing despite 2 weeks of amoxicillin-clavulanate. Please assess antibiotic therapy. [ Not comfortable with any virtual care modality ]

Case 3: *52 year-old with right foot diabetic ulcer progressing despite 2 weeks of amoxicillin-clavulanate. Please assess antibiotic therapy.*


You indicated that you are not comfortable with any virtual care modality. Please explain why:

________________________________________________________________

________________________________________________________________

________________________________________________________________

________________________________________________________________

________________________________________________________________

Display This Question:

If For each of the following vignettes, please select the modalities which you be comfortable using... = Case 4: 55 year-old with 1 month history of lower back pain. MRI showing L4/5 discitis/osteomyelitis – please assess for antibiotic therapy. [ Not comfortable with any virtual care modality ]

Case 4: *55 year-old with 1 month history of lower back pain. MRI showing L4/5 discitis/osteomyelitis – please assess for antibiotic therapy.*


You indicated that you are not comfortable with any virtual care modality. Please explain why:

________________________________________________________________

________________________________________________________________

________________________________________________________________

________________________________________________________________

________________________________________________________________

Display This Question:

If For each of the following vignettes, please select the modalities which you be comfortable using... = Case 5: 60 year-old with 2 month history of fever, fatigue and weight loss. Please assess for infectious causes. [ Not comfortable with any virtual care modality ]

Case 5: *60 year-old with 2 month history of fever, fatigue and weight loss. Please assess for infectious causes.*


You indicated that you are not comfortable with any virtual care modality. Please explain why:

________________________________________________________________

________________________________________________________________

________________________________________________________________

________________________________________________________________

________________________________________________________________

Display This Question:

If For each of the following vignettes, please select the modalities which you be comfortable using... = Case 6: 35 year-old with new diagnosis of HIV infection. Please assess for starting anti-retroviral therapy. [ Not comfortable with any virtual care modality ]

Case 6: *35 year-old with new diagnosis of HIV infection. Please assess for starting anti-retroviral therapy.*


You indicated that you are not comfortable with any virtual care modality. Please explain why:

________________________________________________________________

________________________________________________________________

________________________________________________________________

________________________________________________________________

________________________________________________________________

Display This Question:

If For each of the following vignettes, please select the modalities which you be comfortable using... = Case 7: 24 year-old who returned from travel to Honduras two weeks ago with a non-healing ulcer on the leg. Please assess for infectious causes. [ Not comfortable with any virtual care modality ]

Q23 Case 7: *24 year-old who returned from travel to Honduras two weeks ago with a non-healing ulcer on the leg. Please assess for infectious causes.*


You indicated that you are not comfortable with any virtual care modality. Please explain why:

________________________________________________________________

________________________________________________________________

________________________________________________________________

________________________________________________________________

________________________________________________________________

| Page Break |  |
| --- | --- |

Q10 Please consider the following **outpatient referrals of patients recently discharged from hospital who was seen by one of your colleagues in Infectious Diseases**. For the purposes of the following questions, **you are seeing the patient for the first time two weeks after hospital discharge - the patient has completed two weeks of antibiotic therapy**. Assume that there are no patient-specific or technology-specific barriers to accessing or administering care virtually . Unless otherwise specified, assume that the diagnosis has been confirmed and the accompanying microbiology and investigations are available. Please assume that the remuneration for services provided virtually are identical to those provided in-person.

For each of the following vignettes, please select the modalities which you be comfortable using to assess and manage the patient during the first clinical encounter. (Check all that apply)

|  | Telephone (1) | Video-conferencing Platform (2) | Physician-to-physician e-consultation (3) | Not comfortable with any virtual care modality (5) |
| --- | --- | --- | --- | --- |
| Case 8: 50 year-old with Staphylococcus aureus (MSSA) bacteremia complicated by L1-2 vertebral osteomyelitis/discitis. Blood cultures negative on day 3 of therapy. Planned to receive 6 weeks of IV cefazolin with no surgical intervention. (1) |  |  |  |  |
| Case 9: 67 year-old with with left knee prosthetic joint infection due to Enterobacter, status post first of a two-stage revision arthoplasty, planned to receive 6 weeks of oral ciprofloxacin. (2) |  |  |  |  |
| Case 10: 52 year-old with Streptococcus anginosus bacteremia and liver abscess, treated with percutaneous drain insertion (remains in situ). Planned to receive 4 weeks of IV ceftriaxone. (3) |  |  |  |  |
| Case 11: 45 year-old with with left temporal brain abscess. Planned to receive 4 weeks of IV ceftriaxone and oral metronidazole without surgical intervention. (4) |  |  |  |  |
| Case 12: 68 year-old with Enterococcus faecalis bacteremia and native mitral valve infective endocarditis. Blood cultures negative on day 2 of therapy. Planned to receive 6 weeks of IV ceftriaxone and ampicillin. (7) |  |  |  |  |
| Case 13: 42 year-old with recurrent nephrolithiasis and E. coli bacteremia, treated with lithotripsy. Planned for two week course of oral ciprofloaxcin. (6) |  |  |  |  |
| Case 14: 50 year-old with candidemia related to central venous catheter. Ophthalmology examination did not show retinitis. Planned to receive 2 weeks of oral fluconazole. (5) |  |  |  |  |

Display This Question:

If For each of the following vignettes, please select the modalities which you be comfortable using... = Case 8: 50 year-old with Staphylococcus aureus (MSSA) bacteremia complicated by L1-2 vertebral osteomyelitis/discitis. Blood cultures negative on day 3 of therapy. Planned to receive 6 weeks of IV cefazolin with no surgical intervention. [ Not comfortable with any virtual care modality ]

Case 8: *50 year-old with Staphylococcus aureus (MSSA) bacteremia complicated by L1-2 vertebral osteomyelitis/discitis. Blood cultures negative on day 3 of therapy. Planned to receive 6 weeks of IV cefazolin with no surgical intervention.*


 You indicated that you are not comfortable with any virtual care modality. Please explain why:

________________________________________________________________

________________________________________________________________

________________________________________________________________

________________________________________________________________

________________________________________________________________

Display This Question:

If For each of the following vignettes, please select the modalities which you be comfortable using... = Case 9: 67 year-old with with left knee prosthetic joint infection due to Enterobacter, status post first of a two-stage revision arthoplasty, planned to receive 6 weeks of oral ciprofloxacin. [ Not comfortable with any virtual care modality ]

Case 9: *67 year-old with with left knee prosthetic joint infection due to Enterobacter, status post first of a two-stage revision arthroplasty, planned to receive 6 weeks of oral ciprofloxacin.*


 You indicated that you are not comfortable with any virtual care modality. Please explain why:

________________________________________________________________

________________________________________________________________

________________________________________________________________

________________________________________________________________

________________________________________________________________

Display This Question:

If For each of the following vignettes, please select the modalities which you be comfortable using... = Case 10: 52 year-old with Streptococcus anginosus bacteremia and liver abscess, treated with percutaneous drain insertion (remains in situ). Planned to receive 4 weeks of IV ceftriaxone. [ Not comfortable with any virtual care modality ]

Case 10: *52 year-old with Streptococcus anginosus bacteremia and liver abscess, treated with percutaneous drain insertion (remains in situ). Planned to receive 4 weeks of IV ceftriaxone.*


 You indicated that you are not comfortable with any virtual care modality. Please explain why:

________________________________________________________________

________________________________________________________________

________________________________________________________________

________________________________________________________________

________________________________________________________________

Display This Question:

If For each of the following vignettes, please select the modalities which you be comfortable using... = Case 11: 45 year-old with with left temporal brain abscess. Planned to receive 4 weeks of IV ceftriaxone and oral metronidazole without surgical intervention. [ Not comfortable with any virtual care modality ]

Case 11: *45 year-old with with left temporal brain abscess. Planned to receive 4 weeks of IV ceftriaxone and oral metronidazole without surgical intervention.*


 You indicated that you are not comfortable with any virtual care modality. Please explain why:

________________________________________________________________

________________________________________________________________

________________________________________________________________

________________________________________________________________

________________________________________________________________

Display This Question:

If For each of the following vignettes, please select the modalities which you be comfortable using... = Case 12: 68 year-old with Enterococcus faecalis bacteremia and native mitral valve infective endocarditis. Blood cultures negative on day 2 of therapy. Planned to receive 6 weeks of IV ceftriaxone and ampicillin. [ Not comfortable with any virtual care modality ]

Case 12: *68 year-old with Enterococcus faecalis bacteremia and native mitral valve infective endocarditis. Blood cultures negative on day 2 of therapy. Planned to receive 6 weeks of IV ceftriaxone and ampicillin.*


 You indicated that you are not comfortable with any virtual care modality. Please explain why:

________________________________________________________________

________________________________________________________________

________________________________________________________________

________________________________________________________________

________________________________________________________________

Display This Question:

If For each of the following vignettes, please select the modalities which you be comfortable using... = Case 13: 42 year-old with recurrent nephrolithiasis and E. coli bacteremia, treated with lithotripsy. Planned for two week course of oral ciprofloaxcin. [ Not comfortable with any virtual care modality ]

Case 13: *42 year-old with recurrent nephrolithiasis and E. coli bacteremia, treated with lithotripsy. Planned for two week course of oral ciprofloaxcin.*


 You indicated that you are not comfortable with any virtual care modality. Please explain why:

________________________________________________________________

________________________________________________________________

________________________________________________________________

________________________________________________________________

________________________________________________________________

Display This Question:

If For each of the following vignettes, please select the modalities which you be comfortable using... = Case 14: 50 year-old with candidemia related to central venous catheter. Ophthalmology examination did not show retinitis. Planned to receive 2 weeks of oral fluconazole. [ Not comfortable with any virtual care modality ]

Case 14: *50 year-old with candidemia related to central venous catheter. Ophthalmology examination did not show retinitis. Planned to receive 2 weeks of oral fluconazole.*


 You indicated that you are not comfortable with any virtual care modality. Please explain why:

________________________________________________________________

________________________________________________________________

________________________________________________________________

________________________________________________________________

________________________________________________________________

End of Block: Part III: Outpatient Virtual Care Vignettes

Start of Block: Block 4

Q11 Do you have any general comments related to how virtual care is used in your outpatient Infectious Diseases practice?

________________________________________________________________

Q12 Please provide your e-mail address so we can contact you once our study results become available. Your contact information will only be used for this purposes and will not be shared with others. Your survey responses will be analyzed in aggregate and will not be identifiable in any publications.

________________________________________________________________

End of Block: Block 4
